# A database of *in situ* water temperatures for large inland lakes across the coterminous United States

**DOI:** 10.1038/s41597-024-03103-8

**Published:** 2024-03-09

**Authors:** Troy Sorensen, Eamon Espey, John G. W. Kelley, James Kessler, Andrew D. Gronewold

**Affiliations:** 1 School for Environment and Sustainability, University of Michigan, Ann Arbor, 48104 USA; 2 Department of Electrical Engineering and Computer Science, University of Michigan, Ann Arbor, 48109 USA; 3 Coastal Survey Development Laboratory, National Ocean Service, NOAA, Silver Spring, 20910 USA; 4 Great Lakes Environmental Research Laboratory, NOAA, Ann Arbor, 48108 USA

**Keywords:** Hydrology, Limnology, Climate sciences

## Abstract

Water temperature dynamics in large inland lakes are interrelated with internal lake physics, ecosystem function, and adjacent land surface meteorology and climatology. Models for simulating and forecasting lake temperatures often rely on remote sensing and *in situ* data for validation. *In situ* monitoring platforms have the benefit of providing relatively precise measurements at multiple lake depths, but are often sparser (temporally and spatially) than remote sensing data. Here, we address the challenge of synthesizing *in situ* lake temperature data by creating a standardized database of near-surface and subsurface measurements from 134 sites across 29 large North American lakes, with the primary goal of supporting an ongoing lake model validation study. We utilize data sources ranging from federal agency repositories to local monitoring group samples, with a collective historical record spanning January 1, 2000 through December 31, 2022. Our database has direct utility for validating simulations and forecasts from operational numerical weather prediction systems in large lakes whose extensive surface area may significantly influence nearby weather and climate patterns.

## Background & Summary

Accurately representing spatial and temporal variability of lake surface water temperatures in numerical weather prediction (NWP) systems has been shown (particularly for Earth’s largest lakes) to improve short- and long-term forecasts of regional precipitation, air temperature, and surface wind velocity^1–4^. Thus, realistic representation of lake conditions is crucial for the development of the next generation of climate and weather forecast models^5,6^. The database we introduce here was developed to support this advancement by providing *in situ* validation data for a broader project sponsored by the National Oceanic and Atmospheric Administration (NOAA) through its Joint Technology Transfer Initiative (JTTI). The parallel NOAA JTTI project is designed to optimize representation of lake surfaces in the NOAA Unified Forecast System (UFS) by exploring the sensitivity of UFS lake models to alternative lake bathymetric data sets^7^. Specifically, the NOAA JTTI project evaluates potential impacts of a new global lakes bathymetric dataset (GLOBathy) on simulations of lake surface temperature, and temperature depth profiles, in UFS 1-D lake models^8^. It is informative to note that these models are currently operationalized within NOAA’s High-Resolution Rapid Refresh model, or HRRR^9^, which simulates lake physics using a 1-D lake model included in the Community Land Model v4.5^10^ with a 3-km horizontal resolution, and 10 vertical (depth) layers.

Following an iterative *in situ* monitoring platform selection protocol (details below) we obtained near-surface and subsurface lake temperature data from 134 sites across 29 lakes which (through the parallel NOAA study referenced above) can be used to validate HRRR lake model simulations. We solicited and stored temperature data at the highest temporal resolution available, which varies from site to site; at some sites, data is available at sub-hourly resolution and, at others, at relatively coarse (e.g. semi-annual or monthly) resolution. Of the 134 sites in our database, 84 include temperature measurements across multiple depths, allowing for comprehensive validation of HRRR 1-D lake column model simulations.

**Fig. 1:**
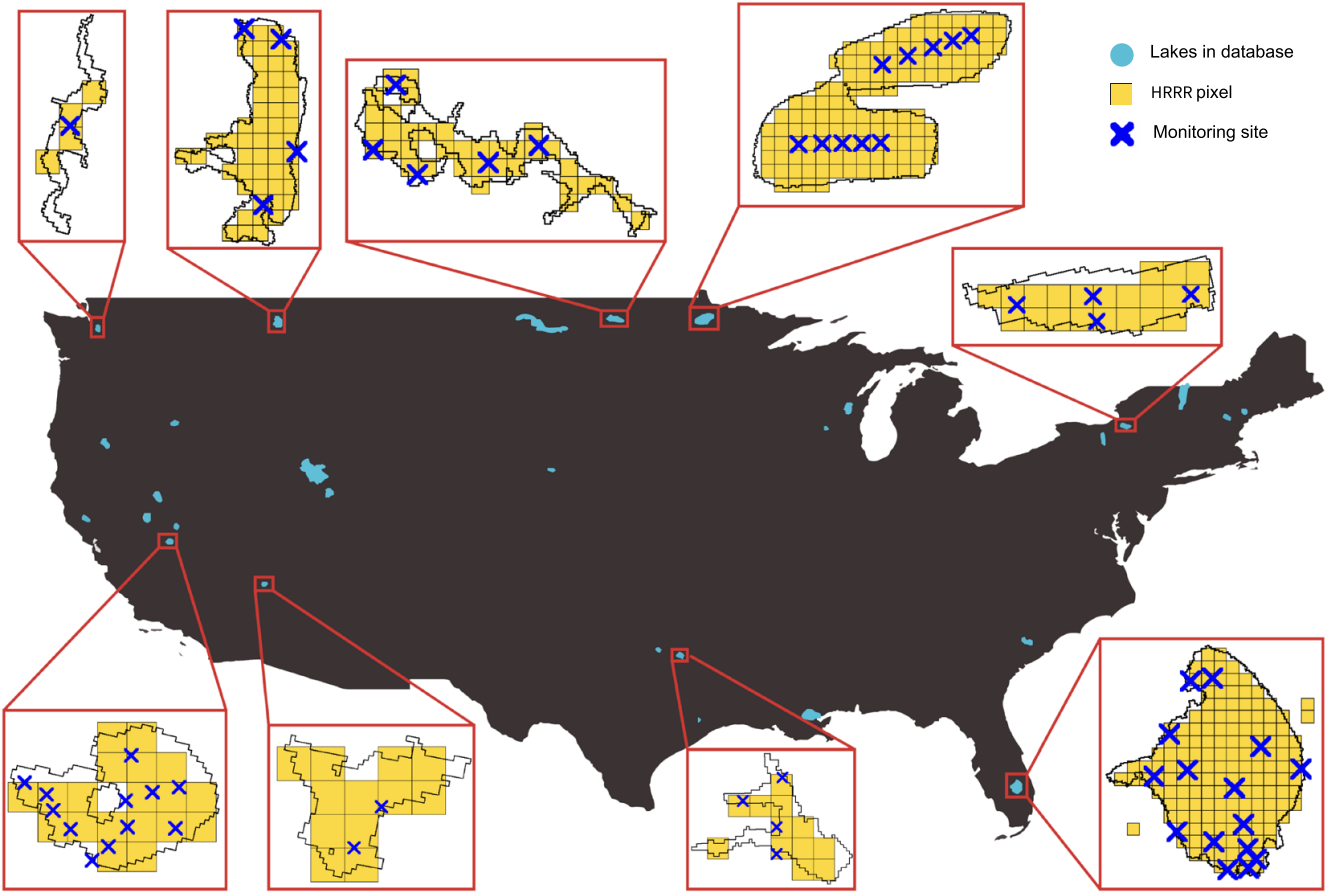
Map of the coterminous United States indicating the location of all 29 lakes in our study (blue polygons; magnified slightly to improve clarity). For 9 representative lakes, we also include an overlay of *in situ* monitoring sites (each represented by a blue ‘x’), lake boundaries, and the corresponding 3 km × 3 km HRRR lake model pixels (yellow grids). The 9 representative lakes are, from top left advancing clockwise, Lake Washington, Flathead Lake, Devils Lake, Red Lake, Oneida Lake, Lake Okeechobee, Lake Tawakoni, Lake Mead, and Mono Lake. See Fig. 5 for corresponding details for all 29 lakes in our study.

**Fig. 2:**
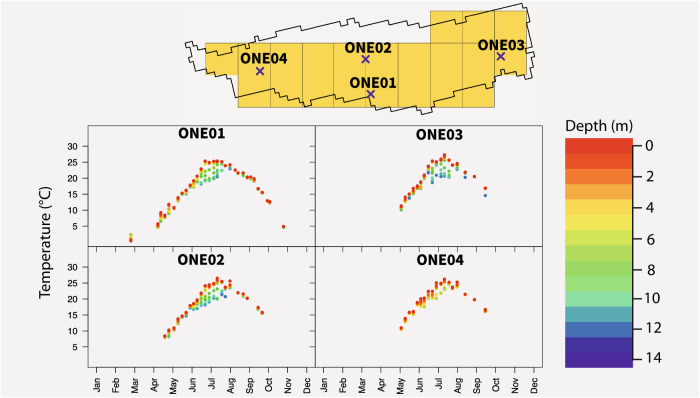
Representative example (from Oneida Lake, NY) of the relationship between *in situ* station locations (top subfigure; each represented by a blue ‘x’ and labeled with its site code), HRRR pixels (yellow squares), and corresponding temperature data for each station in 2019.

While the lakes presented here represent a subset of all lakes in the HRRR model, we believe that, because they are among the model domain’s largest lakes (by surface area; see methods below), they might be expected to have the most profound impacts on surrounding terrestrial weather and climate dynamics. We note that the Laurentian Great Lakes are not included in the NOAA UFS study because they are represented through a separate 3-D modeling framework^11,12^ operated through NOAA’s National Ocean Service (NOS). *In situ* data for validating Laurentian Great Lakes 3-D models is collected and utilized separately and specifically for the NOS modeling initiative, and is therefore not addressed here. Ultimately, the goal of our database is to provide an organized, easily-accessible aggregation of *in situ* lake temperature profile data that can be used not only to support validation for the NOAA UFS 1-D lake model experiments, but to serve as a resource for related lake model validation and empirical data analysis studies as well (see Figs. 1 and 2).

## Methods

**Fig. 3:**
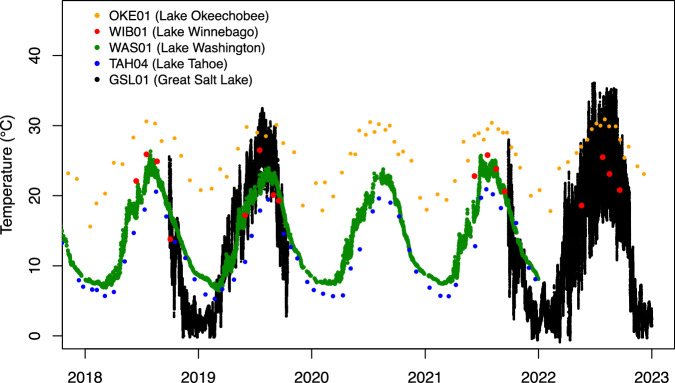
Representative time series (showing only 2018 through 2022 for clarity) of temperature data from five surface (or near-surface) sensors in our database. This time series underscores differences in temporal resolution and continuity across different sensors and lakes; a more comprehensive summary of temporal and spatial (i.e. depth) resolution and continuity is included in Table 2, and the database repository includes a script to visualize the data of each individual sensor. Year labels on x-axis are positioned at the beginning of a calendar year.

We collected lake temperature data from a variety of sources, each requiring a different approach, ranging from scraping online federal agency repositories to collaborating with and soliciting data from local water quality monitoring organizations. Federal agency repositories from which we collected data include the NOAA National Data Buoy Center (NDBC)^13^, the United States Geological Survey (USGS) National Water Information System (NWIS)^14^, and the Water Quality Portal (WQP) - a cooperative service maintained and sponsored by USGS and the United States Environmental Protection Agency^15^. The temperature data we collected from local organizations is unlikely to be included in the aforementioned federal repositories. It is informative to note that any data we have collected for a given lake in our study may be aggregated across one or more of these sources (for a summary, see Table 1). It is also informative to note that data quality, spatiotemporal resolution, and temporal continuity can vary greatly from source to source (see Fig. 3); some sources provide quality-controlled data throughout a lake’s depth profile at high temporal resolution, while others provide relatively sparse temperature data collected by local *ad hoc* or citizen-based groups with little documentation on quality control methods. Feedback from database users has highlighted that direct examination of the data significantly aids in understanding its characteristics, especially for individual lakes or sites. Accordingly, we’ve included a script within the database repository to facilitate the creation of data plots for each site.

**Table 1:** Metadata associated with each lake included in the database.

Lake Name	Latitude	Longitude	Size (km^2^)	Data provider			
				NDBC	NWIS	WQP	Other
Champlain	44.632771	−73.301921	979.6	X	X	X	
Clear	39.048421	−122.808826	136.8			X	
Devils	48.049741	−98.978507	311.7			X	
Flathead	47.893863	−114.130567	463.1				X
Great Salt	41.169322	−112.539431	3962.2		X	X	
Houston	29.975492	−95.141341	22.5		X		
Lewisville	33.121048	−96.979931	51.4			X	
Malheur	43.331553	−118.792309	146.1		X		
Marion	33.484218	−80.315048	228.7			X	
McConaughy	41.248835	−101.792336	66.3			X	X
Mead	36.090574	−114.748057	90.2	X			
Mendota	43.108566	−89.419588	33.8				X
Mono	38.011535	−119.015838	174.1				X
Okeechobee	26.949621	−80.802608	1317.4			X	
Oneida	43.206771	−75.907717	193.7				X
Pontchartrain	30.18727	−90.119927	1655.3	X		X	
Red	48.035195	−94.916255	1123.5			X	
Sakakawea	47.752826	−102.184397	942.1			X	
Sebago	43.861237	−70.551793	105.1				X
Seneca	42.66719	−76.920011	141.2		X		
Tahoe	39.100468	−120.034368	483.8				X
Tawakoni	32.881902	−95.987976	111.2			X	
Upper Klamath	42.428961	−121.934868	271.4		X	X	
Utah	40.220629	−111.824294	321.3			X	
Walker	38.699737	−118.71699	119.2			X	
Washington	47.625051	−122.25055	61.8				X
Winnebago	44.021601	−88.409584	512.4			X	
Winnipesaukee	43.609695	−71.341321	116.6				X

“Latitude” and “Longitude” indicate the center point of the lake and “Size” indicates the surface area as provided by the HydroLakes dataset^27^. The “Data provider” column indicates which data source or sources provided data for each lake; see Table 2 for select summary statistics associated with the data for any given lake and data source.

One of the most important design features of the parallel NOAA lake model simulation study made possible by our database was a focus on evaluating historical lake temperature simulations in 29 of the largest (by surface area) lakes across the continental United States (CONUS). A second important design feature of the NOAA lake model study was a focus on assessing lake model simulation results from just one calendar year (following a model spin-up period) given the relatively high computational expense of running the HRRR model at CONUS scale. Therefore, the collective criteria for including a monitoring platform in our database is that it comes from one of the largest lakes across CONUS for which there is at least one *in situ* temperature observation within a recent calendar year. The selection of a common recent calendar year, in turn, is intended to maximize the total number of temperature data points across the selected lakes and monitoring platforms.

The results of our manual and iterative selection process identified 2019 as the calendar year that maximizes the total number of *in situ* observations across the largest CONUS lakes. Based on our analysis of federal databases and conversations with individual (i.e. local) database managers, it is our understanding that 2019 was (for the purposes of our study) an “optimal” year for aggregating lake temperature data because many *in situ* monitoring platforms were discontinued in 2020 at the onset of the pandemic. As a result of this selection criteria, our database includes data from monitoring platforms for which there was at least one measurement (and, typically, many more measurements) in 2019. However, in order to support any future related empirical and model validation studies we also included any and all data available over a historical period from January 1, 2000 through December 31, 2022, although data availability may vary greatly for years other than 2019 (see Table 2).

**Table 2:** Select summary statistics categorized by data source and then by each lake for which the source has provided data.

Lake Name	Sites	Sensors	Observations	Depth Coverage	Historical Coverage
**NDBC data summary**					
Champlain	3	3	45981	1 m	2019–2020
Mead	2	2	285564	0.5 m	2016–2021
Pontchartrain	1	1	1155458	0.6 m	2008–2022
**NWIS data summary**					
Champlain	1	1	15327	1.5 m	2019–2020
Great Salt	1	1	80214	0.1 m	2018–2022
Houston	1	4	64564	0.9 m–4.3 m	2014–2022
Malheur	1	1	18039	0.5 m	2018–2020
Seneca	1	3	39046	1.8 m–29.6 m	2018–2020
Upper Klamath	1	1	68402	1 m	2007–2022
**WQP data summary**					
Champlain	10	407	4954	1 m–100 m	2000–2021
Clear	6	24	246	0.15 m–10 m	2014–2022
Devils	5	61	228	0.1 m–17 m	2000–2022
Great Salt	4	33	623	0.1 m–10 m	2005–2022
Lewisville	3	46	65	0.1 m–19 m	2006–2022
Marion	7	7	509	0.1 m	2014–2022
McConaughy	1	34	17	0.1 m–33 m	2017–2021
Okeechobee	15	15	1155	0.5 m	2016–2022
Pontchartrain	3	3	305	0.1 m	2008–2021
Red	10	85	1548	0.3 m–9 m	2000–2022
Sakakawea	6	201	431	0.1 m–54 m	2003–2020
Tawakoni	4	56	164	0.3 m–7.9 m	2011–2022
Upper Klamath	6	6	1468	0.1 m	2005–2022
Utah	6	47	457	0.1 m–4 m	2001–2022
Walker	3	52	134	0.1 m–20 m	2006–2022
Winnebago	3	37	197	1 m–21 m	2002–2022
**Summary of data from other sources**					
Flathead	4	4	1864273	0 m	2012–2023
McConaughy	1	32	240	1 m–32 m	2010–2022
Mendota	1	22	20953	0.5 m–20 m	2019–2022
Mono	12	623	553	0.5 m–42 m	2018–2022
Oneida	4	50	1931	0 m–14 m	2000–2020
Pyramid	1	99	76	1 m–99 m	2011–2022
Sebago	1	14	35061	1 m–37 m	2018–2019
Tahoe	4	117	128926	0.5 m–480 m	2010–2021
Washington	1	57	19047	1 m–57 m	2008–2021
Winnipesaukee	1	1	53805	1 m	2016–2022

For each specified data source and lake, “Sites” indicates the number of unique monitoring platform locations (i.e. latitude and longitude), “Sensors” indicates the total number of temperature sensors at any depth across all platforms, “Observations” indicates the number of measurements taken though time across all sensors, “Depth Coverage” indicates the shallowest and deepest sensors, and “Historical Coverage” indicates the earliest and latest years for which data is available.

We used the R Statistical Software (v4.2.1^16^) to extract and store variables from each monitoring platform including sample collection date and time (UTC), coordinate location, depth (m), and water temperature (C). Details on our final data formatting are included in the Data Records section. Details on how we extracted data from each source are included in the subsections below, with related metadata summarized in Tables 1, 2.

### National Data Buoy Center (NDBC)

The NDBC is located within NOAA’s National Weather Service, and is responsible for collecting, managing, and distributing meteorological and oceanographic data from a network of buoys and coastal stations located in oceans, coastal waters, and large lakes (including the Great Lakes, which are not included in this database). All data is quality controlled and publicly available here: (https://www.ndbc.noaa.gov/).

### National Water Information System (NWIS)

The USGS NWIS is a comprehensive database containing a wide range of water-related data including streamflow, groundwater levels, and water quality data including lake temperatures. Lakes that met our study’s criteria were found manually using the online NWIS mapper (https://maps.waterdata.usgs.gov/mapper/), and their data was accessed using the dataRetrieval^17^ package in R by specifying site identification numbers, desired date ranges, and the parameter code for water temperature. Note that the WQP (described in detail below) includes data from the NWIS, but at a much lower temporal resolution.

### Water Quality Portal (WQP)

The WQP is a centralized repository maintained by the United States EPA and the USGS, integrating data from multiple agencies and organizations^18^. Data from sites in the NWIS are included in the WQP, but typically at a lower resolution. Thus data was extracted directly from the NWIS wherever there was overlap with the WQP, and the WQP was instead used to extract data aggregated from other sources.

WQP data can also be accessed in R via the dataRetrieval package, similar to procedures used with the NWIS. Due to the immense amount of data provided by the WQP, users can search for sites that meet certain criteria before requesting a specific site’s data. We used this functionality to search for all sites of type “Lake”, “Reservoir”, or “Impoundment” containing any water temperature data in the year 2019. Once we had a large list of sites meeting this criteria, we used ArcGIS to filter out any sites not located on a lake over 30 km^2^. This left us with a much shorter list of site names which we could then use to query the data of each site individually.

The format for reporting depths of observations varies across different sites within the WQP. Some sites were not given a depth value and were instead reported as “near-surface”; we recorded these as a depth of 0.1 m. Other sites report depth values to a very high precision (<0.1 m). We rounded these depth values to the nearest 1 m for sites containing temperature values throughout a profile of 5 m or deeper, and to the nearest 0.5 m for sites with a shallower profile of less than 5 m. In either case, values of 0 m were then shifted to 0.1 m as the sensors included in the WQP only record bulk temperature.

### Other sources

In addition to the well-known and established described in the sections above, we gathered data from a multitude of other websites and local sources, including the following:

- Flathead Lake - from the Flathead Lake Bio Station (FLBS) site (https://flbs.umt.edu/apps/weather/) which includes surface water temperatures at four sites. All sites have downloadable data dating back to 2011, and we omitted periods of data reporting extremely egregious lake temperature values ( < −50 C).
- Lake McConaughy - provided directly by Nate Nielsen of the Central Nebraska Public Power and Irrigation District (CNPPID). While data at Lake McConaughy is available in the WQP, we were able to obtain higher spatial and temporal resolution data from the files shared with us directly by the CNPPID.
- Lake Mendota - provided online by the Space Science & Engineering Center at the University of Wisconsin-Madison (https://metobs.ssec.wisc.edu/data_download/). We extracted the data at an hourly resolution, though other resolutions are available for download as well.
- Mono Lake - provided directly by Dr. Robert Jellison of the University of California (UC) Santa Barbara and the Mono Lake Committee (https://www.monolake.org/).
- Oneida Lake - provided by Dr. Lars Rudstam at the Cornell University Biological Field Station^19^. While Oneida Lake temperature data is also available through the Knowledge Network for Biodiversity (https://knb.ecoinformatics.org/), the high resolution temperature profile data provided by Dr. Rudstam is not available online.
- Pyramid Lake - provided by Jennessy Toribio, a fisheries biologist at Pyramid Lake Fisheries (PLF). PLF data is sampled monthly, and while there is no exact time specified in the raw data, the PLF stated that readings are typically taken in the late morning. We therefore assigned a timestamp to each recording of 10am local time.
- Sebago Lake - publicly available through the Portland Water District (https://www.pwd.org/sebago-lake-monitoring-buoy).
- Lake Tahoe - provided from two contacts; Dr. Gerardo Rivera at the National Aeronautics and Space Administration (NASA) Jet Propulsion Laboratory (JPL) provided data from three sites at an extremely high temporal resolution, but only for shallow profiles in September 2019. Dr. Shohei Watanabe of the UC Davis Tahoe Environmental Research Center (TERC) provided monthly temperature profiles dating back to 2010 throughout the entire depth profile of Lake Tahoe, but only at a single site. The original TERC data includes temperature measurements at every meter to a depth of 480 m. Our final database includes these temperature measurements at meter intervals to a depth of 50 m, and at 10-meter intervals from 50 m to 480 m (the original higher resolution data is available in the Raw_data section of our database).
- Lake Washington - provided online by King County, WA (https://green2.kingcounty.gov/lake-buoy/Data.aspx). All values from March 2009 were omitted due to noticably incongruous data.
- Lake Winnipesaukee - provided online by the New Hampshire Department of Environmental Services (https://www4.des.state.nh.us/rivertraksearch/search.html).

## Data Records

Our database is deposited in “Deep Blue Data”, the University of Michigan’s institutional data repository^20^. It can be accessed here: 10.7302/7gnd-mj10. The database contains sub-directories for each lake. Within the sub-directory for each lake is the R script used to extract all data for that lake, a metadata table with the latitude/longitude location and depth of each temperature sensor, and a directory containing the temperature data for each sensor in csv format with two columns for the date/time (in UTC) and the temperature (C). Each sensor’s data filename is formatted as ABCXX_YY.csv where ABC is a three letter code for the lake, XX is a unique numerical identifier for the latitude/longitude location of the site, and YY is a numerical identifier for the depth of that sensor. A more detailed explanation of the directory structure is included in a README file within the database.

## Technical Validation

The data we collected from federal agency repositories (e.g. NDBC, NWIS, and WQP) and some local sources were subjected to repository-specific quality control methods, each of which is described in detail in the respective repository’s literature and (if available) web-site. Regardless, we visually inspected all data at all depths and removed data points or time periods periods with egregiously erroneous values for a very small number of sites (as described in the Methods section). Additionally, to ensure overall data reliability, we validated our *in situ* temperatures against remote sensed surface temperature data from the Moderate Resolution Imaging Spectroradiometer (MODIS), which has a spatial resolution of approximately 1 km^21^. We used MODIS Terra and Aqua land surface temperature products, which collectively provide up to four surface water temperature observations per day, per 1-km grid cell, to validate our *in situ* observations.

We recognize there are challenges to comparing gridded remote sensing temperature data to *in situ* data, especially along lake shorelines where there is a potential for land contamination^22^. To address this challenge, we filtered (i.e. removed, prior to validation) *in situ* sites within a 1-km buffer of any lake shoreline, leaving 79 of our total 134 *in situ* sites for validation. Then, because the original intent of our database was to validate (in the parallel NOAA study) HRRR lake models for only the calendar year 2019, we interrogated surface temperature data for MODIS pixels corresponding to each selected *in situ* site location in calendar year 2019 via NASA’s AppEEARS data portal (https://appeears.earthdatacloud.nasa.gov/). Specifically, the products obtained via AppEEARS were MOD21A1D.061^23^, MOD21A1N.061^24^, MYD21A1D.061^25^, and MYD21A1N.061^26^. It is informative to note that the MODIS data obtained through AppEEARS has quality thresholds of 0 (poor), 1 (marginal), 2 (good), or 3 (excellent) for each sensor reading, with each quality threshold corresponding to an error of >2.0 K, 1.5–2.0 K, 1.0–1.5 K, or <1.0 K, respectively. We only used MODIS data with a quality threshold of 2 or higher (i.e. reported error of <1.5 K) for our validation.

**Fig. 4:**
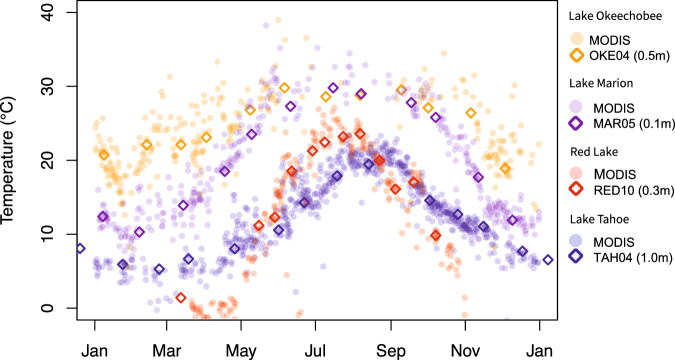
Comparison between bulk surface (or near-surface) and skin temperature data from *in situ* platforms and (respectively) the nearest MODIS pixel at four representative sites across calendar year 2019. Measurement depths for *in situ* platforms are specified in the legend.

A visual comparison between our *in situ* data and data from each corresponding MODIS pixel (see Fig. 4 for a representative time series from four sites) suggests that the *in situ* temperatures are generally consistent with MODIS, with minimal pronounced visible bias. To supplement this visual comparison, we calculated the bias (relative to MODIS) of each *in situ* data point, along with the root-mean-square error (RMSE) of each site and the RMSE across all sites. Specifically, for each MODIS data point, we identified the closest *in situ* value that was collected within 3 hours of the MODIS observation. If there was no *in situ* data collected within 3 hours of the MODIS observation, then that MODIS observation was not used for validation. This approach resulted in 1,808 pairs of *in situ* and MODIS temperatures. The RMSE and bias across all validation data pairs was 2.780 K and 0.023 K, respectively (with MODIS being slightly warmer on average), and a more detailed assessment of RMSE and bias for each monitoring platform (Table 3) indicates that bias is generally low, especially at sites for which there is a high number of observations.

**Fig. 5:**
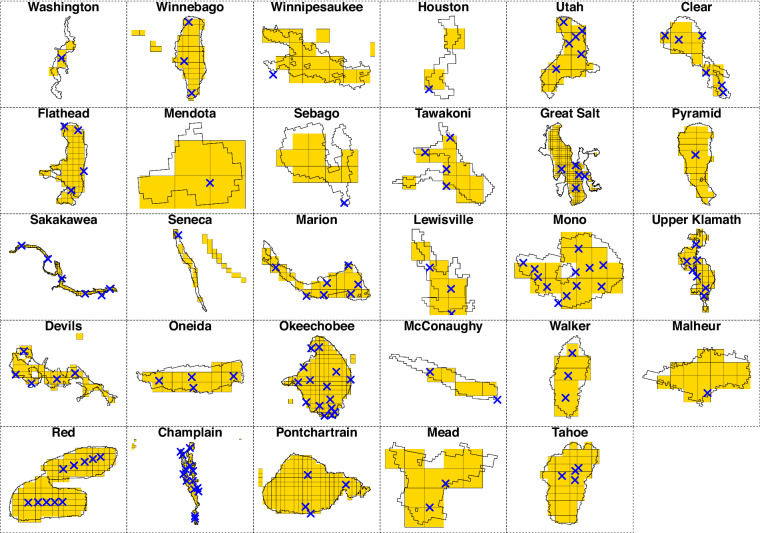
For each lake in our study, a summary of the lake’s shoreline, associated HRRR model pixels (yellow grids; 3 km × 3 km each), and location of *in situ* monitoring sites (represented by a blue ‘x’). Some panels (e.g. Winnebago, Seneca, Champlain) also show HRRR pixels from adjacent water bodies that are not included in our study.

**Table 3:** Summary of root-mean-squared errors (RMSE) and bias (all in C) at each monitoring station based on comparison between *in situ* and MODIS data.

Site Name	Lake	RMSE	Bias	N	Site Name	Lake	RMSE	Bias	N
CHA01_00	Champlain	2.02	−0.32	132	OKE12_00	Okeechobee	1.11	1.11	1
CHA02_00	Champlain	3.53	−0.07	125	OKE15_00	Okeechobee	1.23	1.12	2
CHA03_00	Champlain	2.9	−0.81	123	ONE02_00	Oneida	2.21	−0.28	11
CHA08_00	Champlain	1.93	1.58	9	ONE03_00	Oneida	2.51	0.2	8
CHA09_00	Champlain	0.54	0.18	7	ONE04_00	Oneida	4.14	−1.28	9
CHA10_00	Champlain	2.22	2	6	RED01_00	Red	2.2	0.61	12
CHA11_00	Champlain	3.15	−0.33	5	RED02_00	Red	2.62	0.56	12
CHA12_00	Champlain	1.84	1.06	6	RED03_00	Red	2.23	0.89	11
CHA14_00	Champlain	3.35	−1.09	7	RED04_00	Red	1.65	1.54	10
CLE02_00	Clear	1.52	−1.4	2	RED05_00	Red	1.64	1.51	9
CLE06_00	Clear	1.09	−1.09	1	RED06_00	Red	6.36	−1.58	8
DEV03_00	Devils	1.41	−1.41	1	RED07_00	Red	7.35	−2.23	7
GSL02_00	Great Salt	1.35	0.64	3	RED08_00	Red	1.83	0.6	5
GSL04_00	Great Salt	2.94	−2.65	4	RED09_00	Red	2.58	−0.11	5
GSL05_00	Great Salt	1.93	−1.68	3	RED10_00	Red	1.59	1.27	4
LEW02_00	Lewisville	1.44	−0.03	3	SAK01_00	Sakakawea	0.7	−0.14	2
MAR05_00	Marion	3.05	2.45	3	SAK03_00	Sakakawea	1.13	−1.13	1
MAR06_00	Marion	2.11	2.11	1	SAK04_00	Sakakawea	2.21	0.81	4
MEA02_00	Mead	3.28	0.61	395	TAH01_00	Tahoe	1.53	−0.4	76
MEN01_00	Mendota	3.28	−0.29	200	TAH02_00	Tahoe	1.53	−0.39	78
MON01_00	Mono	4.37	4.15	4	TAH03_00	Tahoe	1.53	−0.61	84
MON03_00	Mono	2.32	−2.24	3	TAH04_00	Tahoe	1.53	−0.13	3
MON04_00	Mono	2.09	−1.7	2	UPK01_00	Upper Klamath	1.53	0.28	350
MON08_00	Mono	2.97	1.39	2	UPK06_00	Upper Klamath	1.53	0.46	8
MON10_00	Mono	2.16	−1.89	3	UTA01_00	Utah	1.53	−1.49	3
MON11_00	Mono	4.21	0.59	5	UTA03_00	Utah	1.53	−1.64	3
OKE02_00	Okeechobee	0.69	0.68	2	UTA04_00	Utah	1.53	−4.08	3
OKE04_00	Okeechobee	1.96	1.63	2	UTA05_00	Utah	1.53	−1.54	3
OKE06_00	Okeechobee	0.45	0.45	1	UTA06_00	Utah	1.53	−1.87	6
OKE07_00	Okeechobee	0.37	0.37	1	WAL03_00	Walker	1.53	−1.4	2
OKE10_00	Okeechobee	1.26	0.98	3	WIN03_00	Winnebago	1.53	3.45	2
OKE11_00	Okeechobee	1.47	1.47	1					

Number (N) of data points used for comparison is included for reference. Only stations for which an RMSE and bias value could be calculated (see methods above) are listed.

## Data Availability

As described in the Data Records section, our database contains the R scripts that we used to extract and format data for each lake. Additionally, the database contains example scripts for organizing and visualizing the data.
